# The Diagnosis of Gastric and Duodenal Ulcers

**Published:** 1911-08

**Authors:** J. Norment Baker

**Affiliations:** Montgomery, Ala.


### THE DIAGNOSIS OF GASTRIC AND DUODENAL ULCERS.

J. Norment Baker, B. A., M. D., Montgomery, Ala

An academic disussion of the multitudinous and multiform phases of this important subject would lead one far beyond the scope of this brief paper; its purport, therefore, shall be to point out the fact that in order to make the diagnosis of gastric or duodenal ulcer, with at least a fair degree of certainty, it is not necessary, nor is it right, to sit idly by and await the concurrence of that old tetrad of symptoms, viz: pain, localized tenderness, vomiting and hemorrhage. True it is, that in the majority of instances, one or more of these symptoms will assert themselves with varying degrees of prominence, but the whole train need not be, in fact, usually is not, present. If we were to make the diagnosis of ulcer in the stomach or duodenum only in the cases in which these classic symptoms manifested themselves in this orderly sequence, certain it is that the major portion of these troubles would pass unrecognized. Further, in reaching conclusions regarding these lesions it does not require an elaborate laboratory equipment. Stomach findings and analyses of feces are sometimes helpful accessories, but are by no means absolutely essential in the reaching of proper deductions.

Ulcers of the stomach and duodenum almost invariably present a definite symptomatology. When located in the duodenal and pyloric areas, or on the lesser curvature near the pylorus, the sympoms are usually fairly clear-cut and simple, and we do not find marked variation in the course of these symptoms. But as the lesion is found farther and farther from the pylorus, these manifestations may, and often do, lose much of their pathognominic significance, and may lead us into error. For example, Moynihan, in a recent article on duodenal ulcer has this to say regarding the diagnosis of this disease. "There are few diseases of which the symptoms appear in such a definite and well ordered sequence as is observed in duodenal ulcer. It is true that there are cases in which the regular appearances of the symptoms is absent or in which one symptom is so exaggerated as to dwarf, or even destroy the value of the others; but these exceptions are few as they do not belittle the value of the general statement that the symptoms of duodenal ulcer are definite and are not easily to be mistaken, and that they appear in an order and with a precision which are indeed remarkable."

For simplicity of study and for clearness in the comprehension of the various symptoms presented, it has seemed not unwise to recognize three stages in ulcer development. It is to be understood that there is no clear-cut line of demarcation between these various stages and that often the one fades imperceptibly into the other, just as is the case in the various forms of syphilis.

In the first stage of ulceraton, which may last for days or weeks, the appetite is invariably good and the nutrition is at par, or even excessive. In fact, the patient will frequently volunteer the statement that he has a keen relish for food and enjoys it. Complaint is made of pain, more or less severe, coming on from one to five hours after eating, when the stomach is empty or is emptying itself. In duodenal ulcer, the onset of pain is delayed longer, usually two to five hours—than in strictly gastric ulcer, because the chief factor in the production of pain is the irritant action of the acids delivered through the pylorus on the open ulcer and is not due to the mechanical friction of the food passing over the inflamed area. This is shown by the fact that anything which neutralizes the acid (Sod-bicarbonate, proteid foods) gives relief. Hence the longer relief experienced by food intake in duodenal as compared with gastric ulcer. Thus we see that more or less complete ease for a greater or less length of time after the taking of food is the rule in these cases, and the heartier the meal, the more pronounced and prolonged will be the comfort experienced. Especially is this noticed in this—the first stage—and when the ulcer is closely pyloric. There is usually more or less gas formation, some vomiting, though by no means always and then not necessarily much, and it may be more of a sour belch with acid, acrid eructations, accompanied by heartburn.

In the duodenal type of ulceration, vomiting is possibly a more prominent symptom than in the gastric form and the time of vomiting is usually at a later period than in the gastric. Moynihan, however, states that vomiting is very infrequent in this lesion until stenosis develops and that the majority of his operated cases have never vomited. Hemorrhage, certainly of any large amounts, is comparatively rare in this stage. This is the most common train of symptoms presented in the earlier stages of ulceration and it is usually repeated daily during the period of attack, with always a certain regularity in the relation to the meals. It is a noteworthy fact that the distress period after food-intake varies but little for different individuals; in fact, it is peculiarly exact for each. (Moynihan.) The physical examination in this first stage is negative and practically without value. The stomach is normal as to size and position; there is usually an excess of HCl, but the stomach contents is otherwise normal. These patients present themselves to be relieved of pain which they claim comes after meals, but which in reality should more properly be classed as pre-meal pain, or, as Moynihan would call it, "hunger-pain." Given the above conditions, lasting for a variable length of time, which may be for days or for weeks, with several intermissions and recurrences, each recurrence being a little sharper and a little longer than its predecessor, and we can then very probably place the lesion in the second stage, as arbitrarily assumed in the beginning of this paper.

In this second stage the appetite may still be good, though perhaps not above normal and we find the patient seizing upon self-instituted dietaries hoping thereby to minimize his discomfort; the pain is pronouncedly more severe and the satisfaction following a hearty meal is markedly less, the pain manifesting itself sooner than in the first stage. Vomiting is more acrid, frequent and of larger quantities, of sour, bitter acid, acrid fluid, which makes the teeth feel as if they were made of chalk. A sense of relief usually follows, for a greater or less period, this vomiting, which may contain blood in small or considerable amounts. Loss of flesh often manifests itself at this stage, which, however, is readily regained during the intermission. Gas and sour eructations are also troublesome symptoms. As a rule no changes in the stomach outlines are yet to be detected. It will thus be seen that this—the second stage—is but an aggravated and exaggerated picture of the first stage.

In the third stage, the desire for food may still remain, but the patient has now become a monomaniac on the subject of enforced starvation and rigid dieting, because of the distress, pain, gas, vomiting sour eructation and bloating. There is practically no relief afforded by food intake and hence the dread of any form of food. Vomiting is frequent and oftentimes large quantities of food which may have been in the stomach for many hours, suggesting serious obstructive lesions at or near the pylorus. The stomach, as a consequence, may be dilated and prolapsed, and succussion or splashing may be present. Constipation, marked in all stages, is especially prominent now. Loss of flesh, emaciation and enemia are pronounced and the patient may have even a cachectic look. Mental depression also is frequent and distressing neurasthenic features are often present. Hydrochloric acid may be normal, lessened, or even absent, though hardly ever, in these later stages, increased. The presence of blood in both macroscopic and microscopic quantities may be frequently seen.

Briefly, these are the chief symptoms manifested by gastric and duodenal ulcers.

This peculiar cycle within the cycle is its most prominent characteristic, and by many able surgeons is considered so pathognomonic as to lead them to ignore the physical findings and to base a diagnosis upon the anamnesis alone. Yet it must be remembered that this type of symptomatology may be approached in gastric hyperacidity, in inflammatory processes of the gall-bladder, in some appendiceal conditions and in chronic inflammatory affections of the pancreas. While this is true, still there are no pathologic conditions that so closely follow this train of symptoms as do ulcers near the pylorus.

In the making of these diagnoses, I know of no one tactor so important as the careful and accurate taking of the history of each individual case. It is the clinician who can properly evolve the real, salient facts and who displays sound judgment and coherent reasoning in arranging—we might almost say, chronologically—the points elicted, who most often will make the most gratifying diagnosis. In studying these ulcer cases, two factors stand forth very prominently—one is their chronicity and the other is their periodicity. The patient rarely fails to relate clearly and precisely that it has been many years since he first began to experience gastric disturbance, mild, perhaps in its beginning, of short duration and but little disturbing to his general health and usefulness; that, as the years have slipped by, in the trouble has been of the same type, has gradually increased in severity until finally he experiences little or no relief. This period of chronicity varies from one to twenty years and often longer. For instance, the average period of years of this chronicity at the Mayo's Clinic is between twelve and thirteen years. The periodicity will also usually be quite clearly brought out by the patient. Early in the disturbance he may say that his "spells of indigestion or dyspepsia," as he terms them, came in the spring or in the fall, or at intervals of three or four or five months, during which periods he felt perfectly well and maintained a normal appetite and digestion. Then his atacks would come on without discoverable cause, suddenly, very often, and continue for days or weeks without interruption. Each day of the attack was a repetition of the preceding and each meal producing the same effect until finally relief came. The periods of attack and of relief alternate at irregular intervals for years and continue for varying lengths of time. The intermissions, that is the periods of relief, gradually decrease, the attacks become more frequent and more severe, until the stomach efficiency is materially crippled by complications such as pyloric obstruction, dilatation, adhesions, and the like. Then it is that the patient fails, at any time, to reach the normal condition and lapses into a chronic dyspeptic or into the third stage of ulcer formation.

Are, then, chronicity and periodicity peculiar to, or limited to, ulcer of the stomach and duodenum?

No. In gall-stones, in some types of appendicitis, in some general disease such as nephritis, more especially the gastric neuroses and hyperchlorhydria, these features stand out with prominence; but it is rather the character of the attack and the peculiar manifestations of the separate accompanying symptoms upon which we must chiefly rely to lead us aright. All four—ulcer, gall-stones, appendicitis and hyperchlorhydria have pain which may be epigastric only; there may likewise also be gas, sour eructations, vomiting complained of in all. It is not the location of pain that tells the story; nor is it the kind of pain, nor its severity, but rather it is (1), the time of pain and other symptoms; (2), the regularity of pain and (3), the means by which the pain or other symptoms are relieved, that give the differential characteristics of these lesions.

In gall-stone disease the pain is totally unrelated to food intake, of a more diffuse and radiating character, sometimes directed backward to the right scapula or downward and outward, along the right costal arch with spasm of diaphragm; of short duration, abrupt onset and equally abrupt disappearance with almost immediate restoration to health. The interim between attacks are perfectly normal, unless there are entangling adhesions or other complications. Unquestionably, the hardest differential diagnosis to make is that between duodenal ulcer and gall-stone disease, especially when the latter is of long standing and the attacks, owing to various complications, are frequently repeated. Indeed, not a few chronic gall bladder patients so complain that stomach lesions appear certain, while in early duodenal ulcer, the symptoms may so clearly simulate gall-bladder trouble as to mislead the most astute.

I recall, some five or six years ago, having a case which very nicely illustrates this perplexing difficulty. A woman, of some thirty years, married, and the mother of several children, rather large and fleshy, had been suffering for some months with periodic attacks of severe pain in the upper abdomen. The attacks nearly always manifested themselves at night, coming on suddenly, lasting for several hours, with dorsal radiation, finally to be relieved only by vomiting or the use of the hypodermic needle. Between the attacks there was some gastric disturbance —principally of gaseous distention and eructations, but it did not appear to be pronounced. This patient was well nourished and had a keen desire for food. The upper abdomen, over the gall-bladder region, was very tender, and the color of her skin, after some dozen or more of these attacks, became quite icteroid. I made the diagnosis of cholelithiasis with probable stone impaction in the common duct and advised operation. The actual findings, at operation, in this case were a well developed ulcer, low down, and a clean bill of health for the gall-bladder and its ducts. Feeling so sure of the diagnosis in this case I was not prepared for a gastro-enterostomy and consequently the operation resolved itself into an exploratory one and did no good.

Both the symptoms and the field of complaint are often almost identical, and it is only by a more careful analysis of all the points brought out above that one will reach the proper conclusions as to the real seat of the trouble.

In these puzzling cases, it is to be remembered that hemorrhage, even in very small or microscopic amounts, would strongly tend to incriminate the stomach or duodenum and to eliminate the bile passages.

Again, there are lesions of a subacute or chronic type in the appendix which, owing to the very intimate association of all parts of the digestive tract, may produce symptoms entirely referable to the stomach. Patients suffering with digestive troubles from this source, present a very diversified and unclassifiable symptomatology, varying from pronounced gastric distress, due most likely to the reflex spasm of the pylorus, to every possible grade of intestinal indigestion—whatever this per se, may be.

Because of its length and its extreme mobility, the free extremty of the appendix is likely to be found in close apposition with or attached to any of the organs of the abdomen. Von Bergman of Riga reports a case in which the patient had suffered for eight months with pain in the epigastrum, fullness and pressure in the stomach, heartburn, loss of appetite and constipation. Pains radiated into the arms and back. The symptoms began from fifteen to thirty minutes after eating and ended frequently with severe vomiting. The vomitus was of sour taste and consisted of undigested food. No blood was present. In the last one and a half months the patient had become much emaciated and weak. A diagnosis of duodenal ulcer had been made by the attending and consulting physicians. On operation the appendix was found to be lengthened, inflamed and attached to the ascending portion of the duodenum, and this with the horizontal part was covered with adhesions. No ulcer or scar was found in the entire course of the duodenum. The adhesions were separated and the appendix removed. A posterior gastro-enterostomy after the method of von Haeckel was made. The patient continued to vomit and died three days later. On post-mortem examination, no ulcer or other pathological change in the stomach or intestines was found.

Not a few of these cases are very perplexing and in order to clear up the situation correctly may require inspection of both appendix and all of the organs of the upper abdominal zone.

And lastly, comes that will-o'-the-wisp, the neurotic, who, of course, will continue to baffle the scientific adjustment of surgical problems, in this, as in every other field.

*Discussion of Dr. Baker's Paper on Practical Points in the Diagnosis of Gastric and Duodenal Ulcers.*

Dr. Northen—Considering the importance of gastric symptoms, this is a subject of importance, and a very valuable paper. Too much attention cannot be given to this subject. In my practice I have found a great many of these patients, and probably had many more suffering where I could not diagnose the case. Think the paper valuable, and hope some of the other doctors will discuss it freely.

Dr. Parke—It is a subject not in my line of work, but one in which I am interested just the same, although some of the points raised I am unable to discuss. The thing that worries me is, that if they are caused from reflex action, I never have been able to figure out why it was when you operated on an ulcer, you can cure the patient. Think this is an important question, and in this day a physician working on the stomach, unless he is a surgeon, has a very restricted line of work. As a matter of fact there is very little to do for these conditions from the fact that most of these cases come from reflex action.

Dr. Baker, in closing—When it comes to the etiology of gastric ulcer, we do not know what causes a gastric ulcer, but the doctor is confusing this trouble with appendiceal trouble, etc.

Gastric ulcer is not caused by reflex trouble, this does not produce the ulcer. This paper was prepared from a physician's standpoint, as gastric and duodenal ulcers are purely medical cases, and should not always be operated on. All of us physicians, as well as surgeons, can diagnose and treat these cases. Then in after years, if there is a return of this trouble, it can be turned over to the surgeon. Most surgeons claim that these cases are very unsatisfactory for operation.

